# The Incidence of Colorectal Cancer Is Decreasing in the Older Age Cohorts in the Zaanstreek Region in the Netherlands: An Age-Cohort Effect

**DOI:** 10.1155/2013/871308

**Published:** 2013-07-07

**Authors:** R. J. L. F. Loffeld, P. E. P. Dekkers, M. Flens

**Affiliations:** ^1^Department of Internal Medicine, Zaans Medisch Centrum, 210 1500 EE Zaandam, The Netherlands; ^2^Department of Gastroenterology, Zaans Medisch Centrum, 210 1500 EE Zaandam, The Netherlands; ^3^Department of Pathology, Zaans Medisch Centrum, 210 1500 EE Zaandam, The Netherlands

## Abstract

*Introduction.* Colorectal cancer (CRC) has a high incidence. Removal of adenomas, the precursor lesion, could be helpful in the prevention of cancer. *Aim.* To investigate the yearly incidence of CRC in consecutive years. *Patients and Methods.* All patients diagnosed with CRC in the years 1990 till 2010 were studied. Date of diagnosis, age at time of diagnosis, gender, and localisation of the tumour were assessed. *Results.* A total of 1575 incident CRC were diagnosed, 865 men (55%) and 710 women (45%). CRC occurred more often in men. In the course of the years, the occurrence of CRC increased. After exclusion of rectal cancer, the percentage of proximal cancer in the colon shows a trend towards increase in the consecutive years. In the twenty consecutive years, the population of the Zaanstreek region increased from 130.000 to 145.330. There was a significant increase of CRC in the age cohort 51–70 in the period of twenty years, while a significant decreasing incidence of cancer was seen in patients above 71 years. *Conclusion.* The decreasing incidence of colorectal cancer in the age cohorts above 71 years possibly reflects indirect evidence of an age-cohort effect due to removal of adenomas in these age cohorts earlier in life.

## 1. Introduction

Colorectal cancer is a disease with a high prevalence, at least in the Western world. It is the third most diagnosed cancer and the second leading cause of death. Several studies clearly show a rising incidence, mostly explained by aging of the population [1, 2]. Evidence in the literature demonstrated that regular screening for colorectal cancer is effective in reducing incidence and mortality [3, 4]. However, screening is not yet widely available.

Colorectal cancer has a well-defined precursor, namely, the adenoma. Removal of adenomas could be helpful in the prevention of cancer. Since adenomas mostly need several years to develop into cancer, and since these lesions tend to occur at younger age, screening for adenomas is recommended above the age of 50 years. Since more than 20 years adenomas are removed presumably endoscopically, it could be expected that the incidence of colorectal cancer should be decreasing. In this time period most patients underwent colonoscopy because of clinical complaints, and adenomas could be detected not only as the cause of, for instance, bleeding but also as innocent bystander not responsible for any complaint.

Statistics do not show a decrease in colorectal cancer incidence. A major problem with population based statistics is that these only report on total numbers in the population and do not correct for age or changes in age cohorts in consecutive years. Also, changes in age of the general population are not taken into account. Hence, any effect of removal of adenomas with respect to the occurrence of colorectal cancer later in life will be camouflaged.

For this reason, a population based study was done in order to investigate the yearly incidence of colorectal cancer in consecutive years. In addition, the age of the general population was also taken into account.

## 2. Patients and Methods

For the present study all patients diagnosed with colorectal cancer in the years 1990 till 2010 in the Zaans Medical Centre, the community hospital of the Zaanstreek region, were studied. In The Netherlands, no screening program for colorectal cancer exists; hence, all patients were diagnosed because of complaints. The records of the pathology department were searched using the MeSH terms: colorectal cancer, colon resection, colon operation, and adenomas with cancer. In addition, these data were cross-referenced with the files of the endoscopy department and the data from the regional cancer registry. From all patients date of diagnosis, age at time of diagnosis, gender, and localisation of the tumour were assessed. Only the first diagnostic procedure from a patient was included. Patients who developed metastases or local recurrences in the course of their disease were excluded from the study. This is because the first presentation of the colorectal cancer was considered as the incident cancer.

The community administration of the city of Zaanstad, capital of the Zaanstreek region, provided details on its population in the years 1990 till 2010. All individuals and patients were put in consecutive age cohorts of ten years in order to gain knowledge on the changes in population in time. On basis of these data trend lines were drawn in MS-office excel files. Statistical analysis was done with chi-square test for contingency tables. A value below 0,05 is considered statistically significant.

The Ethics Committee of the Zaans Medical Centre approved the study.

## 3. Results

**Figure 1:**
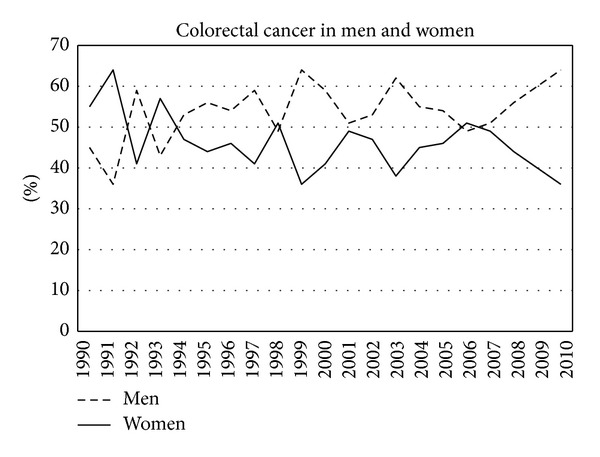
Presence of colorectal cancer in men and women in the consecutive years.

**Figure 2:**
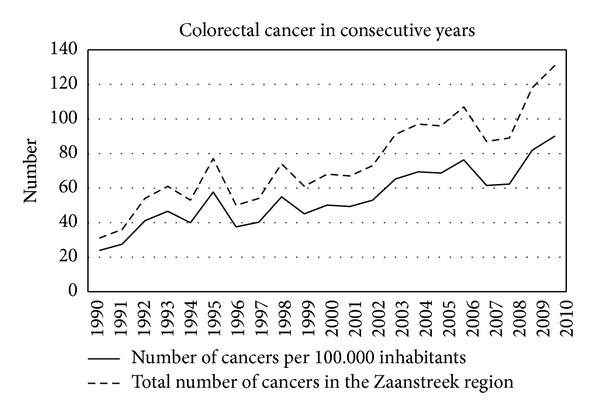
Number of colorectal cancer in the population and per 100,000 inhabitants.

**Figure 3:**
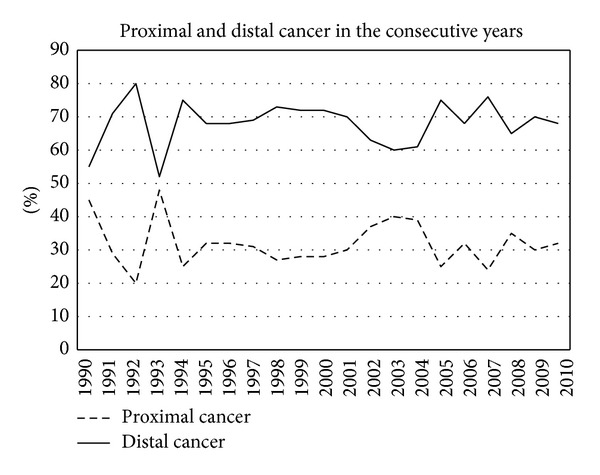
Proximal and distal cancers in the consecutive years.

**Figure 4:**
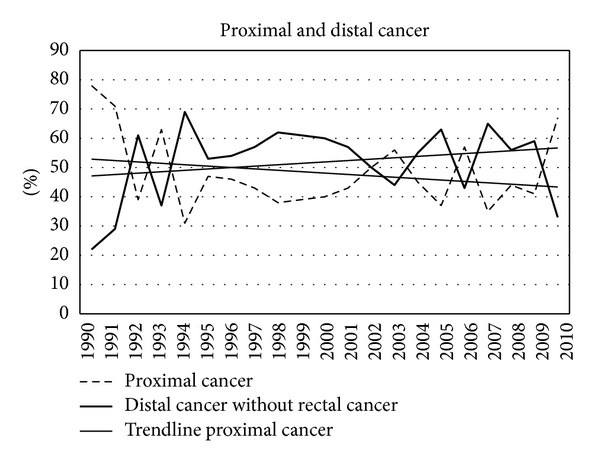
Proximal and distal cancer with exclusion of rectal cancer.

In the study period a total of 1575 incident colorectal cancers were diagnosed, 865 men (55%) and 710 women (45%).  Table 1 shows all diagnosed cancers in the different age cohorts in the consecutive years.  Figure 1 shows the percentage of men and women in the consecutive years. Overall colorectal cancer occurred more often in men.  Figure 2 shows the total number of cancers each year and the number corrected per 100,000 inhabitants. In the course of the years the occurrence of colorectal cancer clearly increased.  Figure 3 shows the localisation of colorectal cancer in the consecutive years. After some fluctuation in the beginning of the nineties the distribution stayed rather constant in the next 16 years. The percentage of proximal cancers, that is cancer located proximal to the splenic flexure, remained lower throughout the years. However, after exclusion of rectal cancer, the percentage of proximal cancer in the colon shows a trend towards increase in the consecutive years (Figure 4).

**Figure 5:**
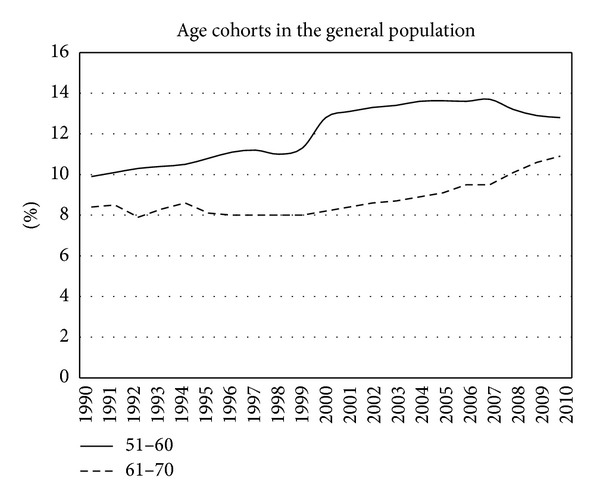
Percentage of age cohorts in the population in the period of twenty years.

In the twenty consecutive years the population of the Zaanstreek region increased from 130,000 to 145,330.  Figure 5 shows the only age cohorts in the general population in the study period that showed a clear increase in the consecutive years reflecting the aging of the “baby boom” generation. The percentage rose from 8,4% to 10,9%.

In Table 1 the absolute numbers of colorectal cancers in each age cohort are shown. 

**Figure 6:**
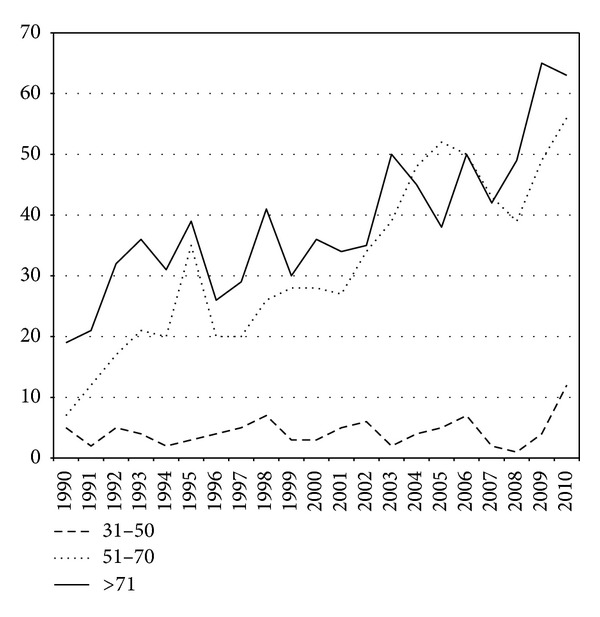
The absolute numbers of colorectal cancer in three age cohorts.

Figure 6 shows the absolute number of colorectal cancer in three major age cohorts. A clear increase in incidence above the age of 50 years is seen. However, Figure 6 shows the same data as percentage of the total population. No changes occurred in the youngest cohort, an increase in the middle cohort, and a decrease in the oldest age cohort. The trend lines show this graphically.

**Figure 7:**
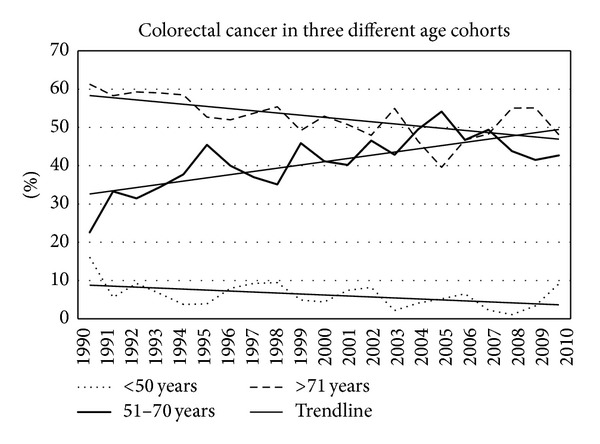
The percentage of colorectal cancer in the total population in the three age cohorts shown in Figure 6.

**Figure 8:**
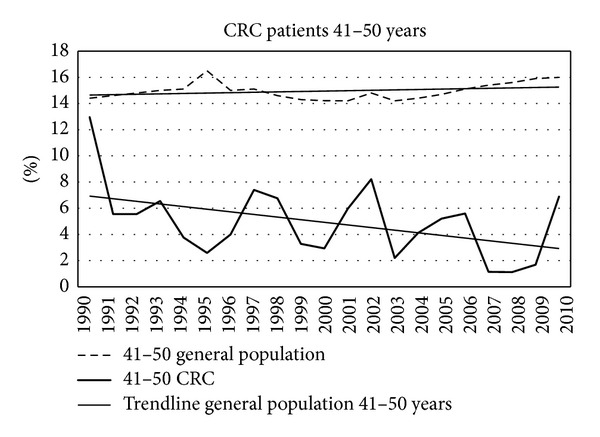
Percentage of colorectal cancers in different age cohorts in consecutive years.

**Figure 9:**
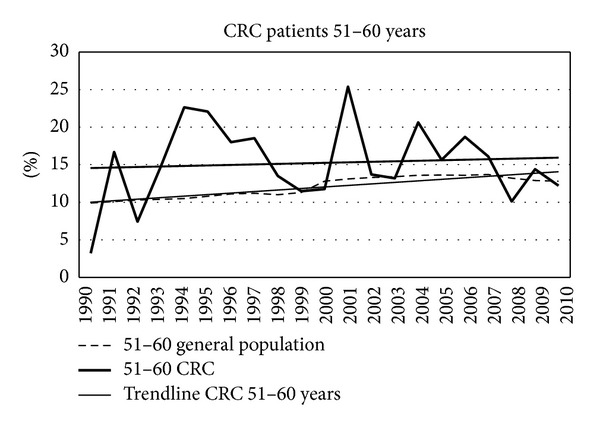
Percentage of colorectal cancers in different age cohorts in consecutive years.

**Figure 10:**
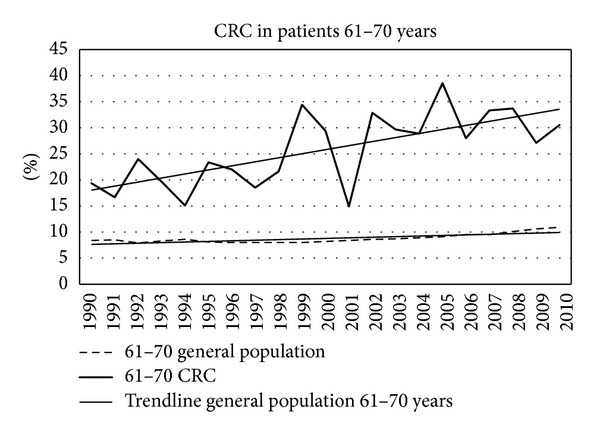
Percentage of colorectal cancers in different age cohorts in consecutive years.

**Figure 11:**
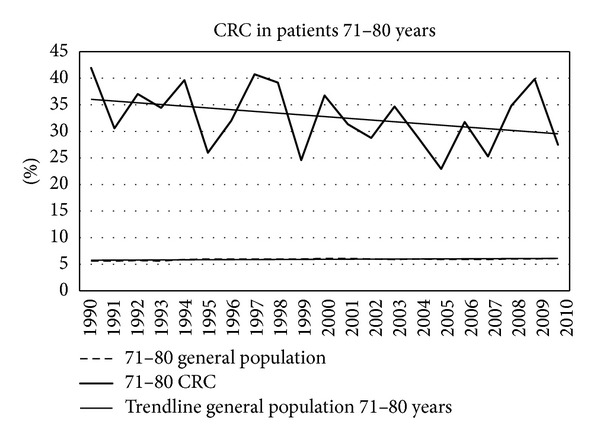
Percentage of colorectal cancers in different age cohorts in consecutive years.

Figures 7–11 show that the percentage of patients with colorectal cancer is each age cohort compared with the total number of people in the same cohort in the general population. The number of cancer in the ages below 40 years was too low to draw a representative figure.  Figure 7 shows the incidence of colorectal cancer in the age cohort 41–50 years. No significant change was noticed in the twenty years. Figures 8–11 show a significant increase of cancer in the age cohorts 51–60 and 61–70 years (*P* = 0.03 and *P* < 0.001 resp.). There was a significant decrease in the age cohort 71–80 years (*P* < 0.001). 

## 4. Discussion

The Zaans Medical Centre is the community hospital of the Zaanstreek region in The Netherlands. It serves all medical care including oncology. Despite the fact that patients can be referred to a university hospital for treatment, the initial diagnosis of colorectal cancer is made in the Zaans Medisch Centrum. Hence, it is acceptable to assume that only a very low number of inhabitants of the Zaanstreek region are initially diagnosed elsewhere. The adherence for gastroenterology is even higher than the total population in the Zaanstreek, due to an endoscopy service without waiting lists. Thus, the patients studied are representative for the occurrence of colorectal cancer at least in the population of the Zaanstreek region. 

The city administration of Zaanstad provided data on the age of the population in the consecutive years.  Figure 5 shows a gradual increase in the age cohort 51–60 and 61–70, that is the “baby boom” generation. Furthermore, the growing population is equally spread in the other age cohorts.

The annual incidence of colorectal cancer in The Netherlands rose from 9239 in the year 2000 to 12319 in the year 2009. This was a gradual increase in the course of these years [5]. The incidence of colorectal cancer in the province of North Holland increased from 45/100,000 in 1990 to 55/100,000 in 2006 [6]. The incidence of cancer in the present study showed a much steeper angle, from 23/100,000 in 1990 to 76/100,000 in 2006. The possible explanation could be the high adherence for endoscopy in the Zaans Medical Centre. There is no screening program for colorectal cancer in The Netherlands. 

Men have higher incidence of colorectal cancer than women [2] in all age groups. In accordance with these regional data and data from the literature, the majority of cancers occur at the age above 55 years [7]. In another study this was above the age of 65 years [2]. 

In the beginning of the nineties the occurrence of proximal cancers, that is proximal to the splenic flexure, showed an increase especially if compared with previous decades [8]. Since 1994 the number of proximal cancers was lower than the number of distal cancers. However, if rectal cancer was excluded from analysis, the percentage of proximal cancers showed a gradual but steady increase. Since most adenomas occur in the sigmoid region, it can be expected that, due to removal of adenomas, more distal cancers are prevented than proximal cancers. 

The most important result of the present small study is the decrease in the incidence of colorectal cancer, corrected for the percentage in the population, in the age cohorts above the age of 71 years. This is despite the fact that the absolute incidence of colorectal cancer is increasing. It is interesting to speculate on the explanation of this decrease. Changes in life style, nutrition, or activities cannot explain this since it can be assumed that changes in the course of many years will occur or apply in all age cohorts. Another explanation is more plausible. Since about 20–25 years colonoscopy is increasingly performed in patients presenting with abdominal complaints, changes in stool, rectal bleeding, or anaemia. In many patients adenomas are detected, often as innocent bystander not responsible for complaints. Subsequently, these adenomas are removed. Since adenomas can be a precursor of cancer later in life, it can be expected that the development of cancer is prevented in these patients. Patients with adenomas are entering endoscopic followup according to local and international consensus. Although interval cancers are described, the occurrence of colorectal cancer in patients under followup is much lower than in people not undergoing follow-up endoscopy [9, 10]. 

The present study suggests a clear age-cohort effect. If in patients belonging to the age cohorts above 71 years polypectomy was done more than twenty years ago, than it can be assumed that cancer was prevented [9, 10]. The fact that the population percentage of colorectal cancer decreases later in life is an indirect circumstantial indication that the expectations are proven. Of course, it is not known how many patients in these older age cohorts had actually undergone polypectomy in the past. A recent study from Germany addressed the question whether removal of advanced adenomas in the colon could actually prevent development of colorectal cancer in the future [7]. The annual transition rates from advanced adenomas to cancer under the age of 65 years appeared to be around 3%, and above the age of 70 years this was 5%. Despite this low transition rate, the number of carcinomas prevented was estimated as 100.000 in total in this screening program. The peak of cancers prevented occurred especially in the age group 70–79 years. The fact that in the present study cancer was decreasing in the older age cohorts in the consecutive years strongly suggests that this age-cohort effect indeed can be the result of removal of adenomas at considerably younger age. The gradual change in subsite distribution of colon cancer is another argument since the majority of adenomas is located between the splenic flexure and the rectum. Removing of adenomas prevents development of distal cancer later in life. The reason why colorectal cancer decreases in incidence in patients below 50 years is not obvious. However, the incidence of cancer in these age cohort is too low to draw any firm conclusions. Possibly this is due to increased awareness.

Although this is a small study with possible confounding, for instance, due to migration of the population, it may be concluded, although circumstantial, that removal of adenomas can prevent development of cancer later in life given the decrease of cancer in patients above 71 years in the period of 20 years. The results of this small study should be confirmed in large nationwide studies in which the age-cohort effect is studied more closely.

## Figures and Tables

**Table 1:** Number of colorectal cancer in the different age cohorts in the consecutive years.

Age cohorts	31–40	41–50	51–60	61–70	71–80	81–90	>91
1990	—	4	1	6	13	6	—
1991	—	2	6	6	11	7	3
1992	2	3	4	13	20	11	1
1993	—	4	9	12	21	14	1
1994	—	2	12	8	21	9	1
1995	1	2	17	18	20	14	5
1996	2	2	9	11	16	10	—
1997	1	4	10	10	22	7	—
1998	1	5	10	16	29	11	1
1999	—	2	7	21	15	13	2
2000	1	2	8	20	25	10	1
2001	1	4	17	10	21	11	2
2002	—	6	10	24	21	14	—
2003	—	2	12	27	31	17	2
2004	—	4	20	28	28	16	1
2005	—	5	15	37	22	15	1
2006	1	6	20	30	34	16	—
2007	1	11	14	29	22	20	—
2008	—	1	9	30	31	17	1
2009	2	2	17	32	47	16	2
2010	3	9	16	40	36	24	3

In 1990, one patient of 28 years was diagnosed with colon cancer; in 1998 and 1999, two patients aged 26 and 30 years were diagnosed with cancer.
